# Tissue Reaction of the Rat Urinary Bladder to Synthetic Mesh Materials

**DOI:** 10.1100/tsw.2009.120

**Published:** 2009-10-02

**Authors:** Gokhan Atis, Serdar Arisan, Aysim Ozagari, Turhan Caskurlu, Ayhan Dalkilinc, Erbil Ergenekon

**Affiliations:** 1st Urology Department, Sisli Etfal Research and Training Hospital, 34377 Istanbul, Turkey

**Keywords:** bladder, mesh, intravaginal slingplasty, tension-free vaginal tape, vypro

## Abstract

The aim of this study was to assess urinary bladder histopathology induced by the sling materials tension-free vaginal tape (TVT), vypro mesh, and intravaginal slingplasty (IVS). Thirty rats were studied: sham-operated controls, TVT, vypro, and IVS groups. After laparotomy, a 0.5- x 1-cm piece of mesh was implanted on the anterior bladder wall. The bladder was examined histopathologically after 12 weeks. Inflammation, foreign-body reaction, subserosal fibrosis, necrosis, and collagen deposition were graded. The Kruskal-Wallis and posthoc Dunn tests were used. The sham-operated rats showed no tissue reactions. The TVT, vypro, and IVS groups showed increased inflammation (*p* = 0.006, *p* = 0.031, *p* = 0.001), subserosal fibrosis (*p* = 0.0001), foreign-body reaction (p = 0.0001), and collagen deposition (*p* = 0.0001) as compared to sham. Inflammation was more intense in the IVS group as compared to the TVT and vypro groups (*p* = 0.041, *p* = 0.028). The bladder presented more increased inflammatory response to IVS than the other meshs. This may play a role in the ultimate outcomes or complications from slings.

## INTRODUCTION

The midurethral synthetic sling procedure is the most popular method for treating stress urinary incontinence and gives the best long-term surgical outcome[1]. Various autologous, alloplastic, and synthetic materials have been used in these procedures, and each has a different success rate, availability, cost, and associated risk, e.g., infection and erosion[2]. The ideal implant material for these procedures would be affordable, available, and efficacious, and have the lowest complication rate possible[3]. Also, it would not cause donor site morbidity, or induce either infection or rejection[4]. Based on pore size, the most common synthetic meshes can be classified into four types[5]. Type 1 is macroporous mesh with pore size greater than 75 µm, type 2 is microporous with pore size less than 10 µm in at least one of their three dimensions, type 3 is macroporus with multifilamentous or microporus components, and type 4 has submicronic pore sizes. Types 1 and 3 meshes are used in pelvic reconstructive surgery, types 2 and 4 meshes are instead used for prostheses such as vascular grafts. Tension-free vaginal tape (TVT) (Ethicon, U.S.) is a nonabsorbable, monofilament, polypropylene mesh (type 1 mesh) that is currently in widespread use, and gives high success and low complication rates[6,7]. Vypro mesh (Ethicon, U.S.), made of multifilament, nonabsorbable, polypropylene and absorbable polyglactin mesh (type 3 mesh), has been shown to produce low-level inflammation because of its large pore size[8]. Intravaginal slingplasty (IVS) (Tyco Healthcare International, U.S.) is made of multifilament polypropylene and is also a nonabsorbable mesh (type 3 mesh).

Animal experiments show that synthetic mesh induces an initial inflammatory reaction that is regulated by the chemical nature of the implanted material, its physical features (e.g., the size of the surface in contact with the recipient tissue), and the mechanical characteristic of the implant[9]. To date, nothing has met the criteria of an ideal prosthetic material.

Accordingly, the aim of the present study was to assess local tissue reactions of the bladder wall to three different synthetic sling materials, TVT, vypro mesh, and IVS, in an experimental rat model.

## MATERIALS AND METHODS

This experimental protocol was approved by the Institutional Animal Care and Use Committee, DETAM, at the Istanbul University Medical Faculty, Istanbul, Turkey. The study was done following accepted guidelines for the care and use of laboratory animals for research. Thirty male Spraque-Dawley rats were randomly placed into four groups: sham-operated control (group 1, n = 6), TVT (group 2, n = 8), vypro mesh (group 3, n = 8), and IVS (group 4, n = 8). Properties of tested biomaterials are listed in Table 1. The animals were anesthetized with an intramuscular injection of ketamine 50 mg/kg. Their abdomens were then shaved and painted with povidone/iodine solution, and a laparotomy was performed under aseptic conditions to access the bladder. Following this, a 0.5- × 1-cm piece of mesh was implanted directly on the anterior wall of the bladder using 5/0 polypropylene sutures at the four corners. The same surgical intervention was applied to each control rat without mesh placement. The abdominal wall was closed with 4/0 polygactin sutures, and the skin was closed with 4/0 catgut sutures. The rats had a standard recovery in the animal care facility. In each group, the rats were killed by decapitation at 12 weeks after surgery, and the bladder of each animal was removed. The interval of 12 weeks was chosen because adverse tissue reactions to sling materials usually occur within the first 3 months in patients undergoing sling surgery[10,11].

**TABLE 1**
**Properties of Biomaterials[17,18]**

| | TVT | Vypro | IVS |
|---|---|---|---|
| Material | Monofilamentous polypropylene | Combined multifilamentous polypropylene, and multifilamentous polyglactin | Multifilamentous polypropylene |
| Mesh type | Type 1 | Type 3 | Type 3 |
| Company | Ethicon | Ethicon | Tyco Healthcare |
| Mesh width (mm) | 10 | 14.2 | 8.3 |
| Pore size (mm) | 1 | 5 | 0.1 |
| Tensile strength (N) | 77 ± 3.7 | 100 ± 1.4 | 50.8 ± 3.4 |

For histopathological examination, specimens were fixed in 10% buffered formalin solution and embedded in paraffin. Sections (5 µm) were prepared and stained with hematoxylin and eosin to define tissue reactions caused by the sling material under light microscopy. All specimens were analyzed histopathologically by the same pathologist blinded to sample origin. Care was taken to avoid including the sutured corners in the analysis to preclude influencing the results by possible tissue reactions to the suture material. Tissue reactions were graded histologically, using a modified numerical scale following the literature[12]. Inflammatory cell infiltration, fibroblast proliferation, collagen deposition, foreign-body granuloma, and necrosis were graded on a 5-point scale: 0 (absence), 1 (occasional presence), 2 (slightly distributed), 3 (abundance), and 4 (confluence of cells or fibers). The depth that the mesh material penetrated into the muscular layer of the wall was also evaluated by dividing the muscularis propria into thirds and noting the depth of penetration in each anterior wall specimen[6].

Differences in histological grading were evaluated according to the Kruskal-Wallis test followed by the posthoc Dunn test performed for multiple comparisons. These data were expressed as medians and range of extremes. In these tests, $p < 0.05$ was considered significant.

## RESULTS

All animals remained alive throughout the experimental course. Histopathological examination of the sham group showed no bladder wall reactions. Significantly, more inflammatory cells (most of which were neutrophils) were found in the TVT, vypro, and IVS groups as compared to the sham group (Kruskal-Wallis test and posthoc Dunn test, $p = 0.006$, $p = 0.031$, and $p = 0.001$, respectively). The inflammatory reactions, including proportion of giant cells, were more intense in the IVS group as compared to the TVT and vypro groups ($p = 0.041$ and $p = 0.028$, respectively) (Fig. 1). Subserosal fibrosis, foreign-body type reaction, and collagen deposition were also increased in all synthetic mesh groups as compared to the sham group ($p = 0.0001$ for all variables).

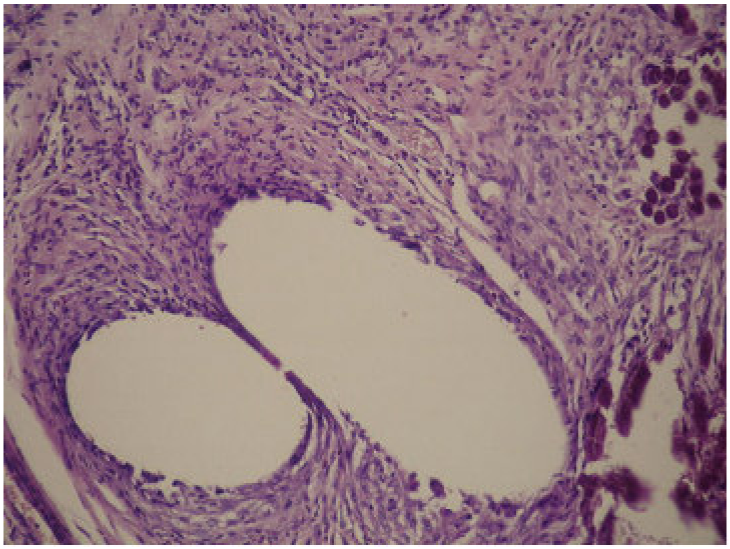

**FIGURE 1.** Inflammation and fibrosis around the IVS.

However, there were no statistically significant differences among the TVT, vypro, and IVS groups with respect to the grades of subserosal fibrosis, foreign-body type reaction, and collagen deposition. Muscle penetration and necrosis were not different among the groups (Fig. 2). For an overview, all data are presented in Table 2.

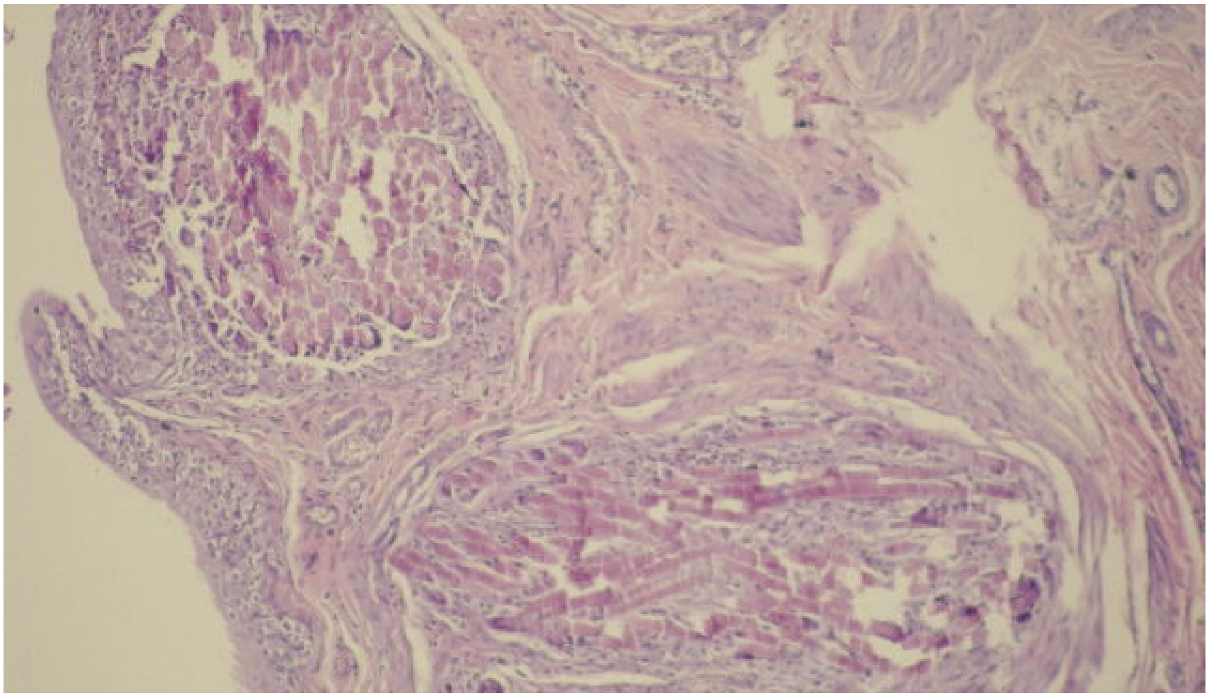

**FIGURE 2.** Muscle penetration into the TVT material.

**TABLE 2**
**Histopathological Grading of Tissue Reactions among the Groups**

| | Sham Group (n = 6) | TVT Group (n = 8) | Vypro Group (n = 8) | IVS Group (n = 8) | *p** |
|---|---|---|---|---|---|
| Inflammation | 0 (0–0) | 1 (0–2)[a,e] | 1 (0–2)[b,f] | 2 (1–3)[c] | 0.0001 |
| Granulocytes | 0 (0–0) | 2 (1–2) | 1.5 (1–2) | 2.5 (2–3) | 0.0001 |
| Macrophages | 0 (0–0) | 1.5 (1–2) | 2 (1–2) | 2 (1–3) | 0.0001 |
| Lymphocytes | 0 (0–0) | 1 (1–2) | 0.5 (0–2) | 1.5 (1–2) | 0.0001 |
| Fibroblasts | 0 (0–0) | 0.5 (0–1) | 0.5 (0–2) | 1 (1–2) | 0.0001 |
| Subserosal fibrosis | 0 (0–0) | 2 (1–3)[d] | 2 (2–3)[d] | 2 (2–3)[d] | 0.0001 |
| Foreign-body type reaction | 0 (0–0) | 1 (1–2)[d] | 1 (1–2)[d] | 1 (1–2)[d] | 0.0001 |
| Collagen deposition | 0 (0–0) | 2 (1–3)[d] | 2 (1–2)[d] | 1.5 (1–2)[c] | 0.0001 |
| Muscle penetration | 0 (0–0) | 0.5 (0–3) | 0 (0–1) | 0 (0–2) | 0.1 |
| Necrosis | 0 (0–0) | 0 (0–1) | 0 (0–0) | 0 (0–0) | 0.12 |

*Note*: Data were given as median and ranges; *Kruskal-Wallis test and posthoc Dunn test; $^{a}p$ = 0.006, $^{b}p$ = 0.031, $^{c}p$ = 0.001, and $^{d}p$ = 0.0001, when compared to the sham group, and $^{e}p$ = 0.041, $^{f}p$ = 0.028, when compared to the IVS group.

## DISCUSSION

Various materials have been used in pubovaginal sling procedures for treating stress urinary incontinence, but there is no consistency in reports on the ideal mesh material. The supporting materials in use include autografts, such as rectus fascia and fascia lata; synthetics, such as polypropylene; and commercially processed cadaver tissues, such as dermis and fascia lata. All types of mesh cause tissue reactions after they are implanted.

Our study was conducted in order to compare the tissue reactions in the rat bladder wall after contact with three different prosthetic materials used in the treatment of stress urinary incontinence. In this study, two types of polypropylene meshes, including monofilament TVT and multifilament IVS, and vypro mesh, a combination of polypropylene and polyglactin mesh, were chosen. Some authors have suggested that the use of mesh with less polypropylene, larger pores, and added polyglactin should prevent severe inflammation[6]. Synthetic meshes were placed in direct contact with the bladder because its histological features are similar to those of the urethra. Yildirim et al. found time-dependent histologic changes, up to day 30 after implantation in the rabbit, to be similar when TVT, IVS, and suprapubic arc sling (SPARC) were compared[4]. In contrast, Krause et al., evaluating tissue reaction to meshes 3 months after implantation in the rat, found IVS to be associated with a more severe inflammatory reaction and a higher degree of fibrosis than TVT or SPARC[13]. Bafghi et al. compared the efficacy and complications between a polypropylene monofilament tape (TVT) and a polypropylene multifilament tape (IVS)[14]. Their results show that the polypropylene multifilament tape caused greater rates of dysuria and infection. Bacterial infection of the multifilament might be explained by the "pore theory", which states that if the pore size (distance between the different filaments of a multifilament mesh) is less than 10 µm, then bacteria (about 1 µm) can hide and proliferate in the mesh, whereas immune cells, such as macrophages (16–20 µm) and leucocytes (9–15 µm), cannot. This would allow the bacteria to escape from the immune cells and colonize the mesh. In our study, inflammatory reaction was found to be increased in the IVS group compared to the other mesh groups. The increased inflammatory and fibrotic response of giant cells may be because of the multifilamentous polypropylene components of the IVS.

Samli et al. investigated bladder tissue reactions to mesh materials. They fixed mesh directly on the bladder wall and reported that there was more marked penetration into the muscularis propria with prolene mesh than with either vypro or vicryl mesh[9]. It is interesting that there was penetration of mesh into the muscularis propria of the bladder at 14 days. In the present study, we did not find any statistically significant difference among the IVS, TVT, and vypro mesh groups with respect to muscle penetration.

Studies of different kinds of synthetic meshes that were implanted in animals to repair hernia showed that an inflammatory reaction caused by the inserted material is followed by the increased development of collagenous tissue. Bogusiewicz et al. evaluated the deposition of collagen in the vicinity of monofilament TVT and multifilament IVS polypropylene tapes implanted in female rats[15]. In their study, the total amount of collagen deposited around the polypropylene mesh implanted in female rats was similar for TVT and IVS meshes, but differences were noted in the arrangement of the collagen fibers and the intensity of the inflammatory reaction. In our study, increased collagen deposition was found in all synthetic mesh groups as compared to the sham group; however, the grade of collagen deposition was not different among the synthetic mesh groups. It is uncertain, however, whether these findings have any impact on either the biomechanical properties of the implant tapes or the efficacy of suburethral sling procedures.

Klinge et al. studied foreign-body reaction to mesh materials and showed a persistent foreign-body reaction that is independent of implantation time, but varies according to the type of material[16]. In the present study, foreign-body type reactions were found in all synthetic mesh groups as compared to the sham group; however, foreign-body type reaction grade was not different according to the type of synthetic mesh materials.

In conclusion, the most important tissue reactions induced by the sling materials were inflammatory response and fibrosis. The rat bladder wall had an increased inflammatory response to IVS as compared to other synthetic mesh materials, such as TVT and vypro mesh. Although mesh may be made from similar materials, the final weave of the product may play a role in the ultimate outcomes or complications from slings. Further studies in animal or human models may be necessary in order to determine the importence of these biomechanical properties.
